# Role of Intra-articular Piroxicam in the Temporomandibular Joint After Arthrocentesis for Anterior Disc Displacement Without Reduction

**DOI:** 10.7759/cureus.34580

**Published:** 2023-02-03

**Authors:** Asmita Gupta, Iqbal Ali, Mohammad Zeeshan, Sudheer Singh, Alok Kumar, Amina Adil

**Affiliations:** 1 Department of Oral and Maxillofacial Surgery, Amarnath Hospital, Varanasi, IND; 2 Department of Oral and Maxillofacial Surgery, Career Postgraduate Institute of Dental Sciences and Hospital, Lucknow, IND; 3 Dentistry, Mahamaya Rajkiya Allopathic Medical College, Saddarpur, IND; 4 Oral and Maxillofacial Surgery, Shyam Hospital, Lucknow, IND; 5 Oral and Maxillofacial Surgery, Laxmi Multispeciality Dental Care and Implant Centre, Prayagraj, IND; 6 Oral and Maxillofacial Surgery, Ahsan Maxillofacial Surgery Center, Jabalpur, IND

**Keywords:** temporomandibular joint, reduction, intra-articular, anterior disc displacement, arthrocentesis

## Abstract

Study design: This is comparative experimental research to evaluate the role of piroxicam in the temporomandibular joint (TMJ) after arthrocentesis.

Objective: To evaluate the role of intra-articular piroxicam in the temporomandibular joint after arthrocentesis for anterior disc displacement without reduction.

Material and methods: Twenty-two individuals (twenty-two TMJs) were evaluated clinically and radiographically for the study, and then they were randomly assigned to one of two groups. As for group I, they were given arthrocentesis using Ringer's solution (100 ml). Group II received an intra-articular injection of 20 mg/mL of piroxicam (in 1 mL of Ringer's solution) after arthrocentesis (100 mL). The same individuals were assessed both before and after surgery to determine the degree to which their symptoms had improved. Patients were seen in the clinic once a week for the first month after surgery, then once a month for the next three months.

Result: Group II patients presented with better results when compared with Group I.

Conclusion: It can be concluded that installing a 1 ml intra-articular injection of piroxicam at a concentration of 20 mg/ml after arthrocentesis improves the relief of symptoms, both qualitatively and quantitatively. Relief of TMJ symptoms reduced the anxiety in the patients as evaluated by the BAIS (Beck’s Anxiety Inventory Scale) score.

## Introduction

Temporomandibular joint (TMJ) disorders (TMD) is a term that encompasses a number of overlapping conditions. It occurs in approximately 10% of the population, with a predisposition toward younger females. The etiopathogenesis of these conditions remains unclear, with mechanical, functional, chemical, and behavioral factors considered to play variable roles. Temporomandibular internal derangement is caused by various factors, including intra-articular obstruction due to temporomandibular joint anterior disc displacement with reduction and temporomandibular joint anterior disc displacement without reduction, in which the disc gets anteriorly displaced and hence resulting in restricted mouth opening and TMJ pain. Other factors include the change in the viscosity of the synovial fluid and the vacuum effect in the synovium [1,2].

Arthrocentesis is a safe, minimally invasive procedure that can be done easily on an outpatient basis. It is mostly done in patients not benefiting from conservative treatment (i.e., occlusal splint, physical therapy, etc.). The lytic mechanism of arthrocentesis washes out the inflammatory mediators such as interleukins and cytokinins, which are responsible for TMJ pain. The relief of pain after arthrocentesis allows an improvement in mouth opening and range of mandibular motion [1].

## Materials and methods

Before commencing the study, prior consent was obtained from the institutional review board (CPGIDSH/583/17). Patients who presented to the Department of Oral and Maxillofacial Surgery with a diagnosis of temporomandibular anterior disc displacement without reduction and a need for minimally invasive arthrocentesis lavage were selected for this study, and informed consent was obtained according to the inclusion and exclusion criteria listed below. The sample size was calculated using the number of TMJ pain patients with a 95% prevalence rate and the use of G-Power software (Heinrich Heine University Düsseldorf, Düsseldorf, Germany). The following statistics were calculated in the present analysis: arithmetic mean, standard deviation, chi-square test, unpaired student t-test, Mann-Whitney 'U' test, and test of significance (p-value < 0.05).

**Figure 1:**
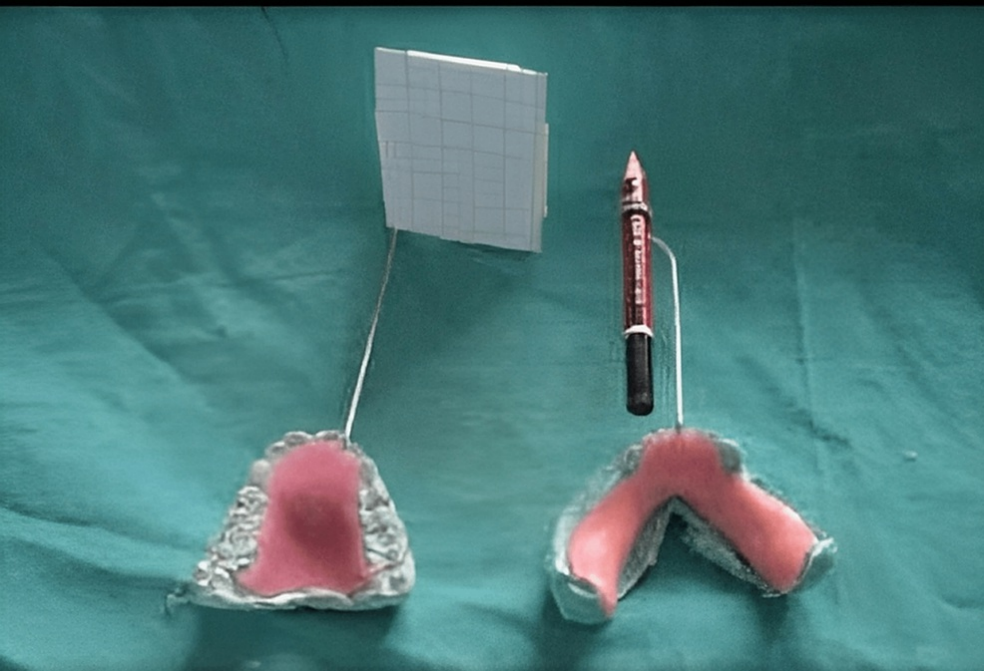
Appliance used for the study

The inclusion criteria include healthy individuals between 16 and 65 years of age, patients who had a closed TMJ lock, patients with restricted mouth opening (25 mm) without any attributable factors, patients with recurrent and painful TMJ clicking, and patients with myofunctional pain dysfunction syndrome (MPDS) (not benefiting from a previous treatment). Patients with uncontrolled systemic conditions such as hypertension, diabetes mellitus, seizures, and so on; pregnant or lactating mothers; acute infections (symptomatic) - either local or systematic; involvement of soft or hard tissues; and patients on disease-modifying anti-rheumatoid agents are all excluded. A thorough clinical and radiological evaluation of the patients was done to identify any criteria that would exclude the patient from the study and, additionally, the fitness of the patient to undergo the procedures. The following pre-operative evaluations were done as part of the clinical assessment: pain on the visual analog scale (VAS) scale, pre-operative measurement of mouth opening, evaluating the TMJ click (present or absent), and maxillo-mandibular models and appliances: construction and graphic plot. A self-constructed appliance for recording the jaw movements - maxillo-mandibular impressions - of the patients was taken and a cast was prepared. Wire bending was done (using the cycle spokes) in such a manner that the pencil could be attached to the lower jaw and graph paper could be attached to the upper jaw (graphic recording could be done during jaw movement). An acrylic plate was made over the wire bending for stabilization and retention in the jaw (additional wire bending was also done so that proper retention and stabilization of the appliance are not disturbed during jaw movement recordings and there is less manual error). Figure 1 shows the appliance for recording lateral excursive movement and protrusive jaw movement.

Patient anxiety scale (Beck’s anxiety inventory scale)

In 1988, Beck et al. [1] developed a 21-item multiple-choice self-report assessment to assess the degree to which adults and adolescents suffer from anxiety. The emotional, physiological, and cognitive symptoms of anxiety are reflected in Beck’s anxiety inventory (BAI) items, but those of depression are not. The BAI consists of 25 questions, each of which is a brief description of a symptom of anxiety. These symptoms may be classified as subjective, neurophysiological, autonomic, or pain-related. The clinician assigns the following values to each response (Table 1).

**Table 1:** Scoring criteria of the scale

Not at all	0
Somewhat	1
Quite	>2
Very	>3

By adding all the points from the 21 separate symptoms, we get a total score, which may be anywhere from 0 to 63. The following scales may be used to measure anxiety levels: 0 to 7 is minimal; 8 to 15 is mild; 16 to 25 is moderate; and 26 to 63 is severe. Radiographic assessment: TMJ open and closed mouth views; magnetic resonance imaging (MRI) for disc and retrodiscal tissue (if required) (Table 2).

**Table 2:** Distribution of patients

Group	Method	No.	%
Group 1	Arthrocentesis using Ringer’s solution	11	50
Group 2	After performing an arthrocentesis using Ringer's solution, a 20 mg/ml piroxicam solution will be injected into the affected joint, in a volume of 1 ml	11	50

Twenty-two patients had clinical and radiological data collected and randomly assigned to one of two groups (group 1 or group 2) over the course of the trial. The randomization was done based on a computer-generated list. Arthrocentesis with Ringer's solution (100 ml) was used to treat this group I. Group II was treated with a 1 ml intra-articular piroxicam injection of 20 mg/ml after arthrocentesis using Ringer’s solution (100 ml). The same individuals were assessed both before and after surgery to determine the degree to which their symptoms had improved. All patients were clinically followed up on a weekly basis for the first month postoperatively, then monthly for the next three months. All the patients in both groups were treated under local anesthesia (2% lignocaine with 1:80,000 adrenaline) by giving an auriculo-temporal nerve block on the affected site. The patients were shifted to the operating theater (OT) and seated at a 45° angle with their heads turned to the contralateral side. The external auditory meatus was blocked with the ointment soframycin soaked in cotton pellets. Site preparation was done with an antiseptic solution, and sterile tape and sterile drape were used to isolate the surgical area. Patient draping was done. Two points were marked over the skin (according to the method suggested by McCain 1988) over the affected joint on the canthotragal line (the posterior entrance points were located along the canthotragal line 10 mm from the middle of the tragus and 2 mm below it). The anterior point of entry was placed 10 mm farther along the line and 10 mm below it).

The access to the posterior recess of the superior compartment of the temporomandibular joint was being treated. The oral and maxillofacial surgeon started the procedure. About 0.5 cubic centimeters of local anesthetic in the form of a mixture of 2% lignocaine and 1:80,000 adrenaline was used to first obstruct the auriculo-temporal nerve. Using an antero-medial-prevalent course and an 18-gauge needle, we may locate the greatest possible joint space at the posterior aspect of the articular prominence (A point). Until there is needle bounceback associated with mandibular growth, just a few milliliters of Ringer's solution are injected latently into the joint. A second needle, placed slightly anterior to the primary needle at point B, is inserted into the TMJ joint area to facilitate the influx of irrigant from the predominant joint space. Patients in group I had arthrocentesis with Ringer's solution (100 ml), whereas those in group II underwent the same procedure but also had the out-streaming needle removed (at point B, for instance) and an intra-articular infusion with 20 mg/ml piroxicam positioned. In the period of three months of follow-up, patients complaining of missing molars, bruxism, and supra-eruption were treated with removable partial dentures (RPD), splints, or grinding, depending on the symptomatic treatment required by them for occlusal correction. In the period of three months of follow-up, patients complaining of missing molars, bruxism, and supra-eruption were treated by RPDs, splints, and grinding depending on the symptomatic treatment required by them for occlusal correction.

Descriptive statistics were used to summarise the findings and compare the two treatment methods across a range of variables. The mean, standard deviation, and percentages were used to summarise the discrete (categorical) data (standard deviation). The chi-square test was used to ascertain whether there was a connection between the two categories of data. Two means were compared using an unpaired Student t-test to determine statistical significance. Mann-Whitney The U test, which compares continuous or ordinal dependent variables on a scale, was used to evaluate whether there were statistically significant differences between the two groups.

## Results

Patients' mean age was 33.45 years for the whole sample, as shown in Table 3.

**Table 3:** Age distribution of subjects

Group	Mean age (year)	Standard deviations	p-value	
					Group I	30.45	10.2	0.287	
Group II	36.45	15.08							
Overall	33.45	12.88							

In group I, the average patient was 30.45 years old, whereas in group II, it was 36.45 years old. Since there was no statistically significant difference (p = 0.287) in the means of the two groups' ages, the participants chosen were representative of both groups with respect to age. On comparing the mean VAS scores of pain at various follow-ups, it was found that there was no statistically significant difference in the mean VAS score for pain before surgery between the two groups (Table 4).

**Table 4:** Comparison of pain rating between the groups SD: standard deviation

Group	Group-I		Group-II		Mann-Whitney U	p-value
	Mean	SD	Mean	SD		
Pre-operative	5.91	1.300	5.82	1.328	58.000	0.898
Immediate post-operative	1.36	0.505	0.64	0.674	26.500	0.023
1st day	2.30	0.218	1.57	0.389	6.500	0.001
2nd day	2.41	0.503	1.64	0.424	12.500	0.001
3rd day	2.23	0.395	1.68	0.582	28.500	0.034
4th day	3.50	0.536	2.00	0.512	0.500	0.001
5th day	3.09	0.437	1.41	0.392	0.000	0.001
6th day	2.57	0.807	1.34	0.465	8.000	0.001
7th day	2.61	0.393	1.36	0.585	5.000	0.001
8th day	2.43	0.298	1.32	0.582	3.500	0.001
9th day	1.77	0.440	0.70	0.445	5.500	0.001
10th day	1.82	0.420	0.52	0.208	0.000	0.001
11th day	1.70	0.445	0.25	0.224	0.500	0.001
12th day	1.48	0.541	0.27	0.467	7.500	0.001
13th day	1.27	0.586	0.11	0.259	4.000	0.001
14th day	1.11	0.728	0.02	0.075	1.000	0.001
15th day	0.98	0.770	0.00	0.000	0.000	0.001
16th day	0.89	0.736	0.00	0.000	5.500	0.001
17th day	0.66	0.625	0.00	0.000	0.000	0.001
18th day	0.61	0.540	0.02	0.075	7.500	0.001
19th day	0.45	0.557	0.00	0.000	16.500	0.002
20th day	0.32	0.420	0.00	0.000	27.500	0.028
21st day	0.43	0.513	0.00	0.000	16.500	0.002
22nd day	0.23	0.344	0.00	0.000	33.000	0.076
23rd day	0.18	0.276	0.00	0.000	38.500	0.151
24rth day	0.27	0.410	0.00	0.000	38.500	0.151
25th day	0.14	0.377	0.00	0.000	49.500	0.478
26th day	0.20	0.332	0.00	0.000	38.500	0.151
27th day	0.14	0.323	0.00	0.000	49.500	0.478
28th day	0.11	0.234	0.00	0.000	44.000	0.300
29th day	0.11	0.303	0.00	0.000	49.500	0.478
1 month	0.20	0.313	0.00	0.000	33.000	0.076

In the first 24 hours after surgery, group II patients reported considerably less pain than group I patients did on a VAS scale (p = 0.023). One month after surgery, there was no statistically significant difference in the mean VAS score of pain between the two groups. Among group I patients, the average preoperative mouth openness was 24.45 ± 0.93 mm, whereas among group II patients it was 22.64 ± 3.38 mm (Table 5).

**Table 5:** Comparison of mouth opening between the groups SD: standard deviation

Mouth opening	Group I		Group II		Significance	
	Mean	SD	Mean	SD	t-value	p-value
Pre-operative	24.45	0.93	22.64	3.38	1.718	0.101
Immediate post-operative	33.18	1.94	34.36	3.14	−1.062	0.301
1st day	33.27	2.33	35.64	1.91	−2.603	0.017
1st week	34.18	2.04	37.36	4.57	−2.110	0.048
1 month	34.45	2.50	37.45	4.50	−1.931	0.068
3 month	34.36	2.29	37.18	4.64	−1.805	0.086

One day following surgery, the average mouth opening for patients in group I was 33.27 mm, whereas the average mouth opening for patients in group II was 35.64 mm. Among those in group I, the average mouth opening after one month was 34.45 mm, whereas those in group II averaged 37.45 mm. On comparing the protrusive jaw movement between the groups, it was found that patients in group I had a mean protrusive score of 4.55 before surgery, whereas those in group II averaged 5.09 (Table 6).

**Table 6:** Comparison of protrusive jaw movement between the groups SD: standard deviation

Protrusive	Group-I		Group-II		Mann-Whitney U	p-value
	Mean	SD	Mean	SD		
Pre-operative	4.55	0.82	5.09	0.70	38.5	0.151
Immediate post-operative	6.27	0.65	6.36	0.50	57.000	0.847
3 months	6.09	0.54	6.18	0.60	55.500	0.748

The Mann-Whitney U test indicated no statistically significant difference (p = 0.151) between the mean protrusive scores of the two groups. Patients in group I had a mean protrusive score of 6.27, whereas those in group II averaged 6.36 immediately post-operation. Before surgery, group I patients had a mean Pre-operative Lateral Excursion score of 5.36, whereas group II patients had a mean protrusive score of 5.64. Mann-Whitney Neither group's mean protrusive score was significantly different from the other's, as measured by the Mann-Whitney U test (p = 0.562) (Table 7).

**Table 7:** Comparison of lateral excursion jaw movement between the groups SD: standard deviation

Lateral excursion	Group I		Group II		Mann-Whitney U	p-value
	Mean	SD	Mean	SD		
Pre-operative	5.36	0.92	5.64	0.92	51.00	0.562
Post-operative	7.55	0.69	9.09	0.54	7.00	<0.001
3 month	6.73	0.65	8.36	0.81	7.00	<0.001

Immediate postoperatively, the average lateral excursion of patients in group I was 7.55 and that of patients in group II was 9.09. The average amount of lateral excursion differed significantly (p = 0.001) between the two groups, as determined by the Mann-Whitney U test. Three months after treatment began, the average lateral excursion for patients in group I was 6.73, whereas for those in group II, it was 8.36. The Mann-Whitney U test revealed a huge disparity (p = 0.001) in the average lateral extension between the two groups. Pre-operatively, the BAIS was minimal in 90.9% of cases in both groups, and in 9.1% of cases it was mild in both groups. Immediate post-operatively, the BAIS was minimal in 90.9% of cases in both groups, and in 9.1% of cases, the BAIS was mild in both groups (Table 8).

**Table 8:** Comparison of Beck’s Anxiety Inventory Scale (BAIS) score between the groups

BAIS			Group I	Group II	Chi-square	p-value
Pre-operative	Minimal	No.	10	10	0.000	1.000
		%	90.9%	90.9%		
	Mild	No.	1	1		
		%	9.1%	9.1%		
Immediate post-operative	Not required	No.	10	10	0.000	1.000
		%	90.9%	90.9%		
	Minimal	No.	1	1		
		%	9.1%	9.1%		
1st day	Not required	No.	10	10	2.000	0.368
		%	90.9%	90.9%		
	Minimal	No.	0	1		
		%	0.0%	9.1%		
	Mild	No.	1	0		
		%	9.1%	0.0%		
1st week	Not required	No.	10	11	1.048	0.306
		%	90.9%	100.0%		
	Minimal	No.	1	0		
		%	9.1%	0.0%		
1 month	Not required	No.	11	11	NA	NA
		%	100.0%	100.0%		
3 month	Not required	No.	11	11	NA	NA
		%	100.0%	100.0%		

Immediately post-operatively, occlusal corrections were not required in 100% of the cases in both groups. Post-operatively, after one day, the occlusal corrections were not required in 100% of the cases in both groups (Table 9).

**Table 9:** Comparison of occlusal corrections between the groups

Occlusal corrections			Group I	Group II	Chi-square	p-value
Immediate post-operatively	Not required	No.	11	11	NA	NA
		%	100.0%	100.0%		
1st day	Not required	No.	11	11	NA	NA
		%	100.0%	100.0%		
1st week	Not required	No.	9	11	2.200	0.138
		%	81.8%	100.0%		
	Required	No.	2	0		
		%	18.2%	0.0%		
1 month	Not required	No.	7	9	0.917	0.338
		%	63.6%	81.8%		
	Required	No.	4	2		
		%	36.4%	18.2%		
3 month	Not required	No.	9	11	2.200	0.138
		%	81.8%	100.0%		
	Required	No.	2	0		
		%	18.2%	0.0%		

## Discussion

When the anterior disc of the temporomandibular joint is displaced, either with or without surgical intervention, it causes discomfort, limited mouth opening, and dysfunction in the temporomandibular joint [1]. Relationships between these symptoms may be established. Intensified discomfort limits mouth opening, contributing to the dysfunction. Joint lavage, on the other hand, is an excellent method for fixing a number of these issues. By removing inflammatory mediators from the joint, the pain is mitigated, and joint mobility is enhanced by the lysis of adhesions [2] and the reduction of restriction from the anteriorly displaced disc [3]. It also claims to alter the viscosity of the synovial fluid, thereby aiding the translation of the disc and condyle [4]. Additionally, once the inflammatory mediators are washed out, further suppression of the inflammatory process within the joint as well as any injury to the joint due to arthrocentesis need to be addressed. With this in mind, 1 ml of piroxicam was injected and evaluated for its ability to reduce and suppress inflammation and thus pain in the joint. Pain is the main presenting and debilitating symptom [5]. The improvement in pain score once the treatment period and subsequent period are over will have prognostic significance. With this aim, patients were given pre-printed VAS forms to be filled out in the preoperative period as well as during the follow-up period at a six-hour interval. The evaluation of pain scores is presented in Table 2. The reason behind the efficacy of arthrocentesis may also be because of the change in viscosity of synovium [6], which helps glycosaminoglycan present in the disc fibrocartilage attract water, thus allowing the disc or articular cartilage to absorb stress applied to it by deforming or leaking water. Because of the presence of local anesthesia, we believe that this decrease in reading immediately post-operatively has no significant prognostic significance [7]. Pain score evaluated on a subsequent day every six hours was averaged for that day and was tabulated. It was found that group I had a significantly higher pain score as compared to group II, indicating better relief for patients in group II. This trend continued subsequently from the first three days. On the fourth day, the pain score in both groups increased slightly. However, the pain score in group II was still significantly better than in group I. For the first three days, the patient was advised to have analgesics when it was of greater discomfort. The follow-up was done for three months, and till then, there was a significant improvement in pain.

On each subsequent day, the pain score was evaluated, and every six hours it was averaged for that day and tabulated. It was found that group I had a significantly higher pain score as compared to group II, indicating better relief for patients in group II. This pattern continued after the first three days. On the fourth day, the pain score in both groups increased slightly. However, the pain score in group II was still significantly better than in group I. In this study, we cannot attribute a particular reason for this increase in pain score on the fourth day in either group, and this needs further evaluation. Subsequently, improvement in pain scores was seen in both groups until the 21st day, and the reduction in pain scores was more pronounced in group II. The results of Ramesh et al. [8] provide support for this conclusion; they found that the VAS level of pain significantly decreased across all postoperative evaluations. And this finding is in correlation with the findings of Al-Said et al. [9].

The patient in our study complained of limited mouth opening, which typically happens in people with temporomandibular joint internal derangement. TMJ internal disc derangement with or without reduction is one of many potential causes of mouth opening restriction. With the aid of a tool we created, which had one arm with a graph plate attached to the upper jaw and one arm with a pencil attached to the lower jaw, we performed graphic tracing in our study. The range of mandibular motion, including mouth opening, the point of click during functional jaw movement, protrusive jaw movement, and lateral excursive jaw movement, was better documented. Both groups experienced a significant increase in mouth opening immediately following surgery, and these scores were statistically compared. The p-value was 0.30, and the difference was not statistically significant. The improvement in mouth opening can be attributed to the local anesthetic solution used intra-operatively around and within the joint, which blocked the free nerve endings present in the synovial membrane, and, secondarily, to arthrocentesis, which helps release the joint adhesions and enables the easy movement of the joint. In our opinion, the improvement in the reading of immediate post-operative mouth opening would not have a major prognostic significance for intra-articular piroxicam due to the use of the local anesthetic solution and arthrocentesis done before injecting 1 ml of intra-articular piroxicam. Piroxicam is an oxicam NSAID, its plasma half-life has been estimated at 45 hours, allowing for once-daily dosing, with peak plasma concentration occurring two to four hours after oral administration. Piroxicam in a single dose of 20 mg to 40 mg has been shown to produce analgesia with a longer duration of action and has been used for the treatment of rheumatoid.

Arthrocentesis helps in reducing inflammatory mediators present in the synovium of the TMJ, which are the main causative agents for pain and restriction in the mandibular range of motion. Hence, on the basis of statistical findings, we can say that group II was better than group I in terms of improvement in mouth opening. Al-Said et al. [9], in their study, found almost the same result, which is in correlation with the findings of Ramesh et al. [8].

Similarly for protrusive and lateral excursive jaw movements, all patients were evaluated in both groups pre-operatively, immediately after the treatment, and three months post-operatively. Evaluation of protrusive and lateral excursive jaw movement recordings is presented in Table 4. Pre-operatively, protrusive jaw movement and lateral excursive jaw movement were found to be statistically insignificant when matched in both groups (for protrusive jaw movement, p = 0.151, and for lateral excursive jaw movement, p = 0.562). Immediately post-operatively, there was a significant increase in the protrusive jaw movement, and these sores were compared between the groups statistically. The p-value was 0.847, and the difference was statistically insignificant. Arthrocentesis allows for more protrusive jaw movement by removing the disc from the glenoid fossa via a vacuum action in the synovium [8], and local anaesthetic effects used intra-operatively around and within the joint block the free nerve endings present in the TMJ synovial membrane [9,10].

Immediately post-operatively, there was a significant increase in the protrusive jaw movement, and these sores were compared between the groups statistically. The p-value = 0.847, and the difference was statistically insignificant. The improvement in protrusive jaw movement is due to arthrocentesis, which probably eliminates the reversible adhesion of the disc to the glenoid fossa caused by the vacuum effect in the synovium [9] and the local anesthetic effect used intra-operatively around and within the joint, which blocked the free nerve ending present in the synovial membrane of the TMJ [10,11].

Patients with an acute closed lock of TMJ do not have a TMJ sound. The TMJ sound occurs due to an obstruction in the gliding movement of the mandible. In our study, preoperatively, the difference in the number of clicks in both groups was insignificant. The evaluation for TMJ lick is in Table 8. Except for two patients, all cases complained of clicking during mouth opening. This is probably due to the obstruction caused by an anteriorly displaced disc or the vacuum effect in the synovium. The improvement in joint clicking is probably due to arthrocentesis, which causes hydraulic distention in the superior joint space [12]. Hence, on the basis of the statistical finding, both groups got better in terms of joint clicking, but comparing both groups, the statistical difference was insignificant. This result is in agreement with the findings of Ramesh et al. [8] and Al-Said et al. [9]. Other than TMJ pain, restricted mouth opening, a reduction in the range of mandibular motion, stress, and occlusal interferences are contributing factors for TMJ internal derangement. Stress can be measured on various scales, and in our study, BAIS was used [13-20]. This result is supported by the findings of Dworkin [14]. In their study, they stated that in the TMJ problem subgroup, pain was consistently related to psychological scores.

The limitations of the article include the limited sample size and the fact that different factors responsible for TMJ pain were not evaluated. There was bias in the use of the VAS scale, which is very subjective. Some more drawbacks to adding include lack of blinding, injecting Ringer’s solution in the joint space in the control group after arthrocentesis, standardization of cases selected using Wilke’s staging, and clarity about the conservative treatment methods provided to the patient.

## Conclusions

As a first-line treatment for symptom management of TMJ anterior disc displacement with or without reduction, arthrocentesis is a quick, minimally invasive technique with good success rates. Installing a 1 ml intra-articular injection of piroxicam at a concentration of 20 mg/ml after arthrocentesis improves the relief of symptoms qualitatively as well as quantitatively. As evaluated by the BAIS score, relief of TMJ symptoms reduced anxiety in the patients. During follow-up, post-arthrocentesis occlusal discrepancies, if found with the improvement in mandibular movement, need to be addressed for the sustenance of symptomatic relief. There were no untoward sequelae or side effects from arthrocentesis or injecting 1 ml of intra-articular piroxicam (20 mg/ml) into the superior joint space of the TMJ. To back up the results of this study, more research with a larger sample size is needed.
